# Home-based self-collection of biological samples, including vaginal swabs: a mixed methods study for Britain’s fourth National Survey of Sexual Attitudes and Lifestyles (Natsal-4)

**DOI:** 10.1136/sextrans-2024-056386

**Published:** 2025-01-11

**Authors:** Clarissa Oeser, Pam Sonnenberg, Magnus Unemo, Katharine Sadler, Soazig Clifton, Jo Gibbs, Simon Beddows, Rebecca Hamilton, Abraham Roodt, Stephanie Migchelsen, Emily Dema, Alexandra David, Catherine H Mercer, Nigel Field

**Affiliations:** 1 Department for Infection and Population Health, Institute for Global Health, University College London, London, UK; 2 World Health Organization Collaborating Centre for Gonorrhoea and other Sexually Transmitted Infections, National Reference Laboratory for Sexually Transmitted Infections, Department of Laboratory Medicine, Faculty of Medicine and Health, Örebro University, Örebro, Sweden; 3 The National Centre for Social Research, London, UK; 4 Virus Reference Department, Public Health Microbiology Division, UKHSA, London, UK; 5 Blood Safety, Hepatitis, Sexually Transmitted Infections and HIV Division, UKHSA, London, UK; 6 The Doctors Laboratory, London, UK

**Keywords:** SEXUAL HEALTH, INFECTION, Communicable Diseases, Diagnostic Techniques and Procedures, Population Surveillance

## Abstract

**Objectives:**

The decennial National Surveys of Sexual Attitudes and Lifestyles (Natsal) provide general population prevalence estimates in Britain for key sexually transmitted infections (STIs) through biosampling. Since methodological choices can impact acceptability and response rates, we evaluated processes for Natsal-4, including face-to-face and remote interview arrangements, non-return of test results and vaginal swab collection in two pilot studies.

**Methods:**

The pilots were conducted during June to August 2021 and February to March 2022. Participants aged 16–59 years were invited to provide urine samples (cisgender men and trans/gender diverse) or three vaginal swabs (cisgender women; urine was requested if vaginal swabs were declined) following interview. Samples were self-collected at home and posted to the laboratory by the interviewer if the interview was face to face, or by the participant if they preferred to collect the sample later or the interview was remote. Process feedback was collected after the first pilot via qualitative interviews with participants and after both pilots through informal interviewer debriefing.

**Results:**

Of 261 participants interviewed (pilot 1=130; pilot 2=131), 161 (62%) consented to biosampling, of which 129 (49%) provided samples. A sample was received from 78/153 (51%) of women, of whom 60 (77%) provided vaginal swabs and 18 (23%) provided a urine sample. A urine sample was received from 51/108 (47%) cisgender men or trans/gender diverse participants. All samples collected immediately after face-to-face interviews were received (n=77), while 64% of samples from participants consenting to post samples after face-to-face interviews and 60% after remote interviews were received. Process feedback confirmed our methods were broadly acceptable.

**Conclusions:**

We demonstrated that our approach to biosampling and STI testing for a national sexual health survey was reasonably acceptable and feasible in the period coming out the COVID-19 pandemic. Self-collection of vaginal swabs for research, which provide higher testing sensitivity than urine, was feasible and acceptable in a home setting.

## Introduction

 Biosampling as part of sexual health research in the general population is crucial for obtaining prevalence estimates for sexually transmitted infections (STIs) and understanding risk factors associated with infection, as this approach includes individuals who are asymptomatic or not seeking care for their symptoms in addition to those attending services.^1^ However, there are important challenges in undertaking biosampling in community or household settings, including stigma associated with STI diagnoses, concerns about deductive disclosure of sexual activity and acceptability of providing samples outside of clinical settings, which might introduce selection bias. Therefore, feasibility and acceptability of methodological choices need careful consideration.

The decennial National Surveys of Sexual Attitudes and Lifestyles (Natsal, https://www.natsal.ac.uk) have included collection of biosamples since Natsal-2 (1999–2001). Biological data, together with demographic, behavioural and clinical information, have enabled the characterisation of STI population distributions and evaluation of national public health interventions, such as the National Chlamydia Screening Programme^2^ and the human papillomavirus (HPV) vaccination programme.^1^

For Natsal-2 and Natsal-3 (2010–2012), the prevalence of key STIs was estimated through testing of urine samples. For Natsal-3, we established ethical principles for STI testing without returning results to participants and demonstrated feasibility and acceptability in a general population probability sample.^3^ However, participants’ willingness to consent for surveys with biosampling has been declining, possibly reflecting changing societal acceptability,^4^ and it was unclear whether the same approach would be acceptable a decade later.

For Natsal-4, testing was planned for *Neisseria gonorrhoeae*, *Chlamydia trachomatis*, *Mycoplasma genitalium*, *Trichomonas vaginalis* and type-specific HPV . It was proposed to collect vaginal swabs because these offer significantly improved sensitivity, particularly for HPV detection, compared with urine samples,^5^ and allow characterisation of the vaginal microbiome. Studies in the USA have demonstrated overall acceptability of self-collected vaginal swabs for research, achieving return rates of 65% (National Health and Nutrition Examination Survey (NHANES),^6^ with samples collected by participants aged 14–59 years in mobile clinics, 2003–2014) and 84% (National Social Life, Health, and Aging Project (NSHAP),^7^ participants aged 57–85 years self-collected swabs at home, 2005–2006). Similarly, online postal self-sampling diagnostic services in Britain have been shown to be highly acceptable^8^; however, there is limited evidence from Britain on self-sampling as part of a research study. Guidance on research conducted in household settings and including younger age groups is lacking globally.

Given these considerations, and to inform the methodology for Natsal-4, two pilot studies were conducted to establish the feasibility and acceptability of self-collected vaginal swabs and to explore if non-return of results remains acceptable.

## Methods

### Participant recruitment

Since 1990, Natsal surveys have involved identification of households through probability sampling of the general population, from which one eligible participant is randomly selected and approached for a survey interview. Due to the COVID-19 pandemic, in addition to the face-to-face option, remote interviews via phone or video call were piloted for Natsal-4 to enable fieldwork to continue.

Following completion of their (~60 min) interview, participants aged 16–59 years were invited to provide biosamples. They were given an information leaflet (see the online supplemental material) and the opportunity to ask the interviewer questions. Participants were asked for electronic consent (see the online supplemental material) to provide a biological sample, and separately to store their sample for future research. A £5 gift voucher was provided to consenting participants as a token of appreciation in addition to the £20 voucher for the interview.

### Sample collection and postal return

Procedures were in place to provide a sample during the interviewer visit (interviewer posted sample) or after the interview (participant posted sample). Sample collection after the interview occurred either because face-to-face participants preferred this or because interviews were remote. For remote interviews, participants were sent sampling kits by post. These participants were contacted by phone within 1 week to check kit receipt and to prompt sample return if not already completed. For the second pilot, we developed an enhanced script for interviewers to encourage participants to provide samples during the interview visit if possible, and we introduced an automated text message reminder for face-to-face participants who agreed to collect a sample after the interview.

Participants were provided with instructions on how to collect their samples (see the online supplemental material). All cisgender women, including pregnant or menstruating participants and irrespective of sexual experience, were invited to provide three self-collected vaginal swab samples. Those declining vaginal swabs, cisgender men and trans/gender diverse participants were asked to provide a self-collected urine sample using a first-void urine collection device (Colli-Pee, https://novosanis.com).

### Participant and interviewer feedback

Qualitative interviews using a topic guide were conducted with 20 participants from pilot 1, following their participation to understand how the request to collect biological samples was perceived and their overall experience. These were done by telephone or Microsoft Teams and an encrypted audio recording (Amolto, https://amolto.com/). Participants received £30.

After both pilots, interviewers completed a feedback form and 2-hour debrief session to give feedback and discuss their experiences.

## Results

### Sampling

Two pilots were conducted in June to August 2021 and February to March 2022. Of 261 participants interviewed (pilot 1 n=130, pilot 2 n=131; cisgender women n=153, cisgender men n=101, trans/gender diverse n=7), 161 (62%) consented to biosampling. In total, 129 samples were received at the laboratory, consisting of 77/77 (100%) from participants where the interviewer posted the sample, and 52/84 (62%) from participants who posted their own sample after a face-to-face interview or a remote interview.

This meant that a sample was received at the laboratory from 78/153 (51%) of women participants, of whom 60 (77%) provided vaginal swabs and 18 (23%) provided a urine sample after declining vaginal swabs. Among the 75 women not providing a sample, 21 (28%) had consented (17 to a vaginal swab).

Similarly, a urine sample was received from 51/108 (47%) cisgender men or trans/gender diverse participants. Of those who consented, 11/57 (19%) did not provide a sample.

### Process feedback

Qualitative follow-up interviews were conducted with 20 participants (14 women, six men) (table 1). Overall, participants understood that the purpose of the study was to collect research data, rather than to provide sexual health services. Two participants cited the non-return of test results as the reason for declining to provide a sample, while others stated that not receiving results was reassuring, as it meant that their information was anonymous. Participants who were interviewed face to face and opted to collect their sample after the visit gave reasons for this, including not having enough time, finding it uncomfortable and awkward, wanting time to read the leaflet without the interviewer being present and feeling that they could not provide urine at that moment.

**Table 1:** Participant and interviewer quotes from interviews and debrief sessions illustrating motivations for and against providing samples

About providing a sample	Participants	“there was no point in doing half a survey, might as well do all of it.” “….it was needed to advance our medical knowledge.” “It's a very easy way to have extra £5, so - I thought - I'm happy to do it” "(The experience) was OK. I remember feeling just slightly uncomfortable. I mean in terms of pain, not my feelings. There was some physical discomfort in doing it. But it was fairly minimal and it was not dissimilar to the experience of using other kind of products. So, I wasn't anxious about it.” “the most difficult part was taking it to the post office.” “(I am) happy to do whatever, as long as it is not painful”.
	Interviewers	“…surprised at how keen some participants were to give vaginal swabs”. “One woman said she had a menstrual cycle and asked if it was okay and then did it.” “They weren’t embarrassed. One said it was an invasion of privacy but was relaxed and didn’t think it was an issue”
About not providing a sample	Participants	“When we got to it, I was not going to be around for a while I didn’t really want to commit to something I could not deliver because we were going to be away and also to be honest, I don’t really know why I didn’t want to do it. I have done plenty of sexual health tests in the past at the time. I don’t really know what turned me off, but at the time I just didn’t really fancy it. I guess I had put an hour into the study and it is kinda like more commitment and at the time I was under a lot of work pressures and commitments and I just said no.” “…the intimacy of the vaginal sample I just did not feel comfortable doing that” “…felt too stressed with children around and felt it was easier to give urine” “…just felt more comfortable doing urine.”
	Interviewers	“They said they couldn’t be bothered, but no embarrassment” “(The participant) thought (she) was too old at start for most questions. Refused swab and urine for same reasons” “The argument that the swab sample was no more difficult than an LFT did not help convince those who just felt embarrassed at the process.”
About non-return of results	Participants	“Not having the results was probably better because then you’re not waiting for something horrible to come through the letterbox”. “…it's not an STI clinic…there is other resources for that”
	Interviewers	“Majority of respondents said they would have liked the results and a couple of people said they would only do the sample if they were able to get results”

All 18 interviewers attended a debrief and/or completed a feedback form (table 1). Interviewers highlighted participants’ wishes to support research and advance medical knowledge as reasons for providing a sample, while feeling uncomfortable and that the request was intrusive were reasons for declining a sample.

## Discussion

In our pilot studies in 2021 and 2022, overall, 49% of participants provided a biological sample for STI testing, with minimal differences by gender. This compared with 60% in 2010–2012 (Natsal-3)^1^ and 72% in 1999–2001 (Natsal-2).^2^ Although self-sampling and posting of biological samples might have become normalised during the COVID-19 pandemic, our findings are consistent with a wider trend of declining response to population surveys. For biosampling, this may reflect participants’ concerns about privacy and confidentiality (including risks of data linkage and data sharing with third parties), and indicate a need for greater transparency and attention to how data security is presented.^4 9^ There is also some evidence that research participants who provide data and samples increasingly expect to receive test results.^9^ However, our approach, based on previous work, suggests that not returning results with full explanation to participants remains acceptable in a general population study of sexual health in Britain, where sexual healthcare is widely available and free at the point of access.^1 3^

The mode of interview, timing of sample collection and whether samples were returned by interviewers or participants were important and affected consent and receipt of samples. Self-collected vaginal swabs were provided by 39% of those eligible and accounted for 77% of all samples from cisgender women, demonstrating feasibility and relative acceptability for this kind of research in a home setting. Given the improved sensitivity profile of vaginal swabs over urine samples for detecting STIs and better characterisation of the vaginal microbiome, these data were reassuring and suggested that vaginal samples could be collected in Natsal-4.

Our mixed methods findings provided insights about the piloted processes for Natsal-4 at a time of significant uncertainty given the pandemic and wider changes in response rates to population health surveys. As a result, the approach taken forward for Natsal-4 was to request self-collected biosamples without return of results, with options to provide samples with an interviewer present or after face-to-face interview, or following remote interview. Cisgender women were invited to provide vaginal swabs or urine if these were declined.

## Supplementary material

10.1136/sextrans-2024-056386 online supplemental file 1
