# Supplementary material for: Home-based self-collection of biological samples, including vaginal swabs: a mixed methods study for Britain’s fourth National Survey of Sexual Attitudes and Lifestyles (Natsal-4)
Source: Sex Transm Infect. 2025 Jan 11;101(4):e056386. doi: 10.1136/sextrans-2024-056386 (PMC12128757; doi:10.1136/sextrans-2024-056386)
Supplement: online supplemental file 1 [file sextrans-101-4-s001.pdf]

infections at a population level, but they may not be as accurate as the ones used by the NHS to make a diagnosis. Testing through the NHS is the best way to find out (and treat) if you have an infection; (2) we are testing for some infections and harmless bacteria that do not need treatment; (3) not returning results means that we do not need to link your sample to your name and address.

What if I change my mind?

You can withdraw your consent to participate in the study at any point in time by contacting NatCen Social Research using the details on this page. NatCen will inform the laboratory and the sample will be destroyed. We will also delete all data about you that is personally identifiable (e.g. contains information such as your name, address and date of birth), but we will not remove data from de-personalised datasets.

What should I do if I am worried about having a sexually transmitted infection (STI) or about my sexual health?

If you have concerns that you may have been infected with an STI, we suggest you seek professional advice. Free testing for STIs, as well as free confidential advice about sexual health, cervical screening, family planning and contraception, can be obtained online or from your local sexual health or Genitourinary Medicine (GUM) clinic, your family GP, family planning clinics, as well as some pharmacies and youth centres. The following website will allow you to find your nearest sexual health service: **https://www.nhs.uk/service-search/sexual-health/find-a-sexual-health-clinic**

The interviewer will provide you with more information about organisations near you and their telephone numbers; you can also visit their websites for further information.

The UK Health Security Agency (UKHSA - previously part of Public Health England) recommends sexual health check-ups for sexually active people every time they change partner. This is because STIs may be present without a person knowing or having symptoms.

Your co-operation is very much appreciated

If you have any questions, you can contact NatCen Social Research by writing to 35 Northampton Square, London, EC1V 0AX; calling freephone **0800 652 4568** or emailing **natsal@natcen.ac.uk**

Our team is from NatCen Social Research, University College London, the London School of Hygiene and Tropical Medicine and the University of Glasgow.

Natsal

National Study of Health and Relationships (Natsal) 2022

Development Study

Urine sample information leaflet

IRAS 275649_16/11/2021_Urine sample information leaflet_designed_v5

ANNEX N - DR urine sample information leaflet_v7.indd 1-2

25/01/2022 10:24

Thank you for taking part in the interview. We would also like your help with the next part of the study that involves you collecting a biological sample.

We very much hope you will agree to give a sample, as these samples are an important part of the study. However, you don't have to give a sample if you prefer not to.

Why give a urine sample?

We would be very grateful if you would agree to provide the Natsal research team with a urine sample. The sample will be tested for some infections that can be transmitted through sexual activity, as well as other bacteria that are harmless and live in the genital tract. It is important to know how many people have different sexually transmitted infections (STIs) in Britain to help plan for sexual health services. To do this, we need to get a true picture of the levels of infections by asking all participants of the Natsal study if they would be willing to provide a sample for testing.

Who is being asked to take part?

We are asking everyone taking part in Natsal to provide a sample, even if they are not sexually active or think they are at no risk of STIs. It is important that as many people take part as possible, as this will help us understand the true overall picture of STIs in the population of Britain.

What if I have never had sex?

We are still interested in a sample from you if you have never had sex. Your sample helps us understand what is happening in the general population.

What do I have to do?

£5

You will be provided with a kit that contains instructions and a container to collect a small amount of urine. This should be done in a bathroom and will only take a few minutes. If you have a face-to-face interview, you can hand the container back to the interviewer who will pack and post it for you. Otherwise, you will be provided with a pre-paid envelope and instructions about how to pack and post your sample yourself in a Royal Mail postbox. You will be able to ask the interviewer any questions you may have. To thank you for your co-operation, a £5 gift card will be given to everyone who provides a sample.

What will my sample be tested for?

The sample will be sent to a certified laboratory and will be tested for the following:

Chlamydia and Gonorrhoea: These are infections that can be passed on through sexual contact. They can be treated with antibiotics, but if not treated can lead to problems such as infertility and can be passed on to sexual partners.

Human papillomavirus (HPV): This is an infection that can be passed on through sexual contact. HPV can cause genital warts or, in rare cases, can lead to cervical or other types of cancer. A vaccine to protect against common types of HPV is available.

Trichomonas vaginalis: This is an infection that can be passed through sexual contact. It may be more common in women but can also be found in men and can be treated with antibiotics.

Mycoplasma genitalium: This is an infection that can be passed through sexual contact. However, we need more information about how common it is. At present, people are not tested for this infection unless they have symptoms, and detected infections are treated with antibiotics.

Microbiome: This is the term used for all bacteria that live in the human genital tract. We want to know how the microbiome varies by age, gender, and sexual behaviour.

The tests above will **never** involve human DNA or human genetic analysis. Sample collection and storage is controlled by the Human Tissue Act (2004). The samples may be sent to the World Health Organization STI reference centre in Sweden and the Wellcome Trust Sanger Institute in the UK, which are run by Natsal investigators.

What happens to the rest of my sample?

We will ask you separately for consent to store what is left over from your sample after the tests listed above have been done. If you agree, any remaining sample may be used for future studies investigating infections in the population, for example, when new tests become available. Stored samples will only be used for future studies if all necessary approvals have been obtained in advance. As before, any new studies will not involve analysis of human DNA or genetics. The sample will not be labelled with your identifying information and researchers who want to use the stored sample will have to apply for permission. If you do not agree to the rest of your sample being stored for future studies, NatCen will inform the laboratory and it will be destroyed. You may still give a sample even if you do not wish any remaining sample to be stored for future use.

What happens to the results of these tests?

The test results will be linked to the answers given in your interview and the information will be used for research purposes only. The laboratory will not know your name or address. The data will be kept strictly confidential, and no results will ever be traced to you or given to you. All data will be dealt with according to the General Data Protection Regulation (GDPR) 2018 and Data Protection Act 2018. For more information on how the information you provide will be used, please see the privacy information at **natcen.ac.uk/natsal-study/privacy-policy**.

Why will my test results not be returned?

We will not return individual results to you because: (1) the tests are good enough to help us understand what is happening with these

ANNEX N - DR urine sample information leaflet_v7.indd 3-4
25/01/2022 10:24

treatment; (3) not returning results means that we do not need to link your sample to your name and address.

What if I am on my period or menstruating?

We are still interested in samples from you and the tests will not be affected. However, if you prefer, you can collect a sample a few days later when your period has stopped.

What if I am pregnant?

We are still interested in samples from you and there is no risk to self-collecting samples if you are pregnant. The swabs used to collect the samples are small and smooth.

What if I change my mind?

You can withdraw your consent to participate in the study at any point in time by contacting NatCen Social Research using the details on this page. NatCen will inform the laboratory and the samples will be destroyed. We will also delete all data about you that is personally identifiable (e.g. contains information such as your name, address and date of birth), but we will not remove data from de-personalised datasets.

What should I do if I am worried about having a sexually transmitted infection (STI) or about my sexual health?

If you have concerns that you may have been infected with an STI, we suggest you seek professional advice. Free testing for STIs, as well as free confidential advice about sexual health, cervical screening, family planning and contraception, can be obtained online or from your local sexual health or Genitourinary Medicine (GUM) clinic, your family GP, family planning clinics, as well as some pharmacies and youth centres. The following website will allow you to find your nearest sexual health service: **https://www.nhs.uk/service-search/sexual-health/find-a-sexual-health-clinic**

The interviewer will provide you with more information about organisations near you and their telephone numbers; you can also visit their websites for further information.

The UK Health Security Agency (UKHSA - previously part of Public Health England) recommends sexual health check-ups for sexually active people every time they change partner. This is because STIs may be present without a person knowing or having symptoms.

Your co-operation is very much appreciated

If you have any questions, you can contact NatCen Social Research by writing to 35 Northampton Square, London, EC1V 0AX; calling freephone **0800 652 4568** or emailing **natsal@natcen.ac.uk**

Our team is from NatCen Social Research, University College London, the London School of Hygiene and Tropical Medicine and the University of Glasgow.

NatCen Social Research

UCL

LONDON SCHOOL of HYGIENE &TROPICAL MEDICINE

University of Glasgow

Natsal

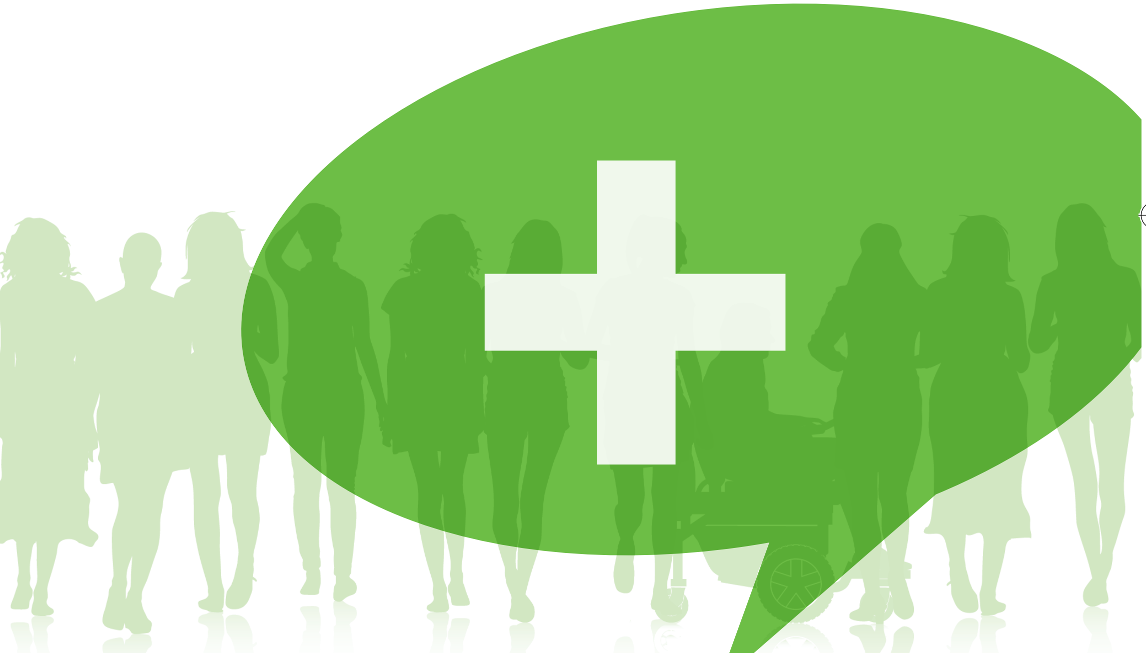

National Study of Health and Relationships (Natsal) 2022

Development Study

Vaginal sample information leaflet

IRAS 275649_16/11/2021_Vaginal sample information leaflet_designed_v5

ANNEX O - DR vaginal sample information leaflet_v7.indd 1-2
25/01/2022 10:24

Thank you for taking part in the interview. We would also like your help with the next part of the study that involves you collecting some biological samples.

We very much hope you will agree to give a sample, as these samples are an important part of the study. However, you don't have to give a sample if you prefer not to.

Why give vaginal samples?

We would be very grateful if you would agree to provide the Natsal research team with vaginal samples using small cotton swabs. The samples will be tested for some infections that can be transmitted through sexual activity, as well as other bacteria that are harmless and live in the genital tract. It is important to know how many people have different sexually transmitted infections (STIs) in Britain to help plan for sexual health services. To do this, we need to get a true picture of the levels of infections by asking all participants of the Natsal study if they would be willing to provide a sample for testing.

Who is being asked to take part?

We are asking everyone taking part in Natsal to provide a sample, even if they are not sexually active or think they are at no risk of STIs. It is important that as many people take part as possible, as this will help us understand the true overall picture of STIs in Britain.

What if I have never had sex?

We are still interested in samples from you if you have not had sex. Your samples help us understand what is happening in the general population. There is no risk to self-collecting samples. The swabs used to collect the samples are small and smooth.

What do I have to do?

You will be provided with a kit that contains instructions and containers to collect vaginal samples. This part of the study involves you inserting a small cotton swab approximately 2 inches (5 cm) into the vaginal opening and gently swirling it around, before placing it into a collection tube. This should be repeated two more times, for a total of three swabs. This should be done in a bathroom or somewhere else private and will only take a few minutes. If you have a face-to-face interview, you can hand the containers back to the interviewer who will pack and post them for you. Otherwise, you will be provided with a pre-paid envelope and instructions about how to pack and post your samples yourself in a Royal Mail postbox. You will be able to ask the interviewer any questions you may have. To thank you for your co-operation, a £5 gift card will be given to everyone who provides these samples.

What will my samples be tested for?

The samples will be sent to a certified laboratory and will be tested for the following:

Chlamydia and Gonorrhoea: These are infections that can be passed on through sexual contact. They can be treated with antibiotics, but if not treated can lead to

problems such as infertility and can be passed on to sexual partners.

Human papillomavirus (HPV): This is an infection that can be passed on through sexual contact. HPV can cause genital warts or, in rare cases, can lead to cervical or other types of cancer. A vaccine to protect against common types of HPV is available.

Trichomonas vaginalis: This is an infection that can be passed through sexual contact. It may be more common in women but can also be found in men and can be treated with antibiotics.

Mycoplasma genitalium: This is an infection that can be passed through sexual contact. However, we need more information about how common it is. At present, people are not tested for this infection unless they have symptoms, and detected infections are treated with antibiotics.

Microbiome: This is the term used for all bacteria that live in the human genital tract. We want to know how the microbiome varies by age, gender, and sexual behaviour.

The tests above will **never** involve human DNA or human genetic analysis. Sample collection and storage is controlled by the Human Tissue Act (2004). The samples may be sent to the World Health Organization STI reference centre in Sweden and the Wellcome Trust Sanger Institute in the UK, which are run by Natsal investigators.

What happens to the rest of my samples?

We will ask you separately for consent to store what is left of your sample after the tests listed above have been done. If you agree, any remaining samples may be used for future studies investigating infections in the population, for example, when new tests become available. Stored samples will only be used in future studies if all necessary approvals have been obtained in advance. As before, any new studies will not involve analysis of human DNA or genetics. The samples will not be labelled with your identifying information and researchers who want to use the stored samples will have to apply for permission. If you do not agree to the rest of your samples being stored for future studies, NatCen will inform the laboratory and they will be destroyed. You may still give the samples even if you do not wish any remaining samples to be stored for future use.

What happens to the results of these tests?

The test results will be linked to the answers given in your interview and the information will be used for research purposes only. The laboratory will not know your name or address. The data will be kept strictly confidential, and no results will ever be traced to you or given to you. All data will be dealt with according to the General Data Protection Regulation (GDPR) 2018 and Data Protection Act 2018. For more information on how the information you provide will be used, please see the privacy information at **natcen.ac.uk/natsal-study/privacy-policy**.

Why will my test results not be returned?

We will not return individual results to you because: (1) the tests are good enough to help us understand what is happening with these infections at a population level, but they may not be as accurate as the ones used by the NHS to make a diagnosis. Testing through the NHS is the best way to find out (and treat) if you have an infection; (2) we are testing for some infections and harmless bacteria that do not need

ANNEX O - DR vaginal sample information leaflet_v7.indd 3-4 25/01/2022 10:24
